# Early warning system models and components in emergency and disaster: a systematic literature review protocol

**DOI:** 10.1186/s13643-019-1211-5

**Published:** 2019-12-06

**Authors:** Hamid Reza Khankeh, Seyed Hossein Hosseini, Mehrdad Farrokhi, Mohammad Ali Hosseini, Nasir Amanat

**Affiliations:** 1 Health in Emergency and Disaster Research Center, University of Social Welfare and Rehabilitation Sciences, Tehran, Iran; 2 Department of Clinical Science and Education, Karolinska Institute, Stockholm, Sweden; 3 Nursing Department, University of Social Welfare and Rehabilitation Sciences, Tehran, Iran

**Keywords:** Early warning system, Model, component, Emergency, Disaster

## Abstract

**Background:**

Emergency and disaster are on the rise in the world. One of the most important components of disaster risk management is the early warning system. Studies have shown divergent models of warning systems with different structures. However, since no systematic review of early warning systems in disasters has been conducted so far, a systematic review of the models, components, and structures of these systems is essential. This protocol is a systematic review study, which aims to evaluate the existing warning systems and their structure.

**Methodology:**

This study attempts to comprehensively search the previous studies with terms and expressions including *disaster*, *emergency model*, *early warning system*, and their synonyms at MESH. To this end, English articles, which have been published from 1980 to 2019, will be assessed. Google Scholar, PubMed, Web of Science, and Scopus databases as well as relevant specialized websites will be searched. Studies will be evaluated by two individuals independently.

**Discussion:**

To the best of our knowledge, no systematic review of models, structures, and components of the early warning system has been conducted so far. This study is the first attempt to comprehensively evaluate the models and components of early warning systems. Accordingly, this study will provide evidence of models, structures and elements of the early warning systems.

**Systematic review registration:**

PROSPERO CRD42018116111

## Background

Emergency and disaster and their consequences are increasing worldwide [1, 2]. The increasing trend of emergency and disaster has changed the paradigm of response to risk prevention and management [3]. One of the most important components of disaster risk management is the advanced early warning system [4]. Upstream documents such as the Hyogo and the Sendai documents have emphasized the importance of the early warning system as one of the key elements of disaster risk reduction. The Sendai document has set out an early warning system with a multi-hazard approach as a requirement for the countries road map by 2030 [5, 6]. The United Nations Office for Disaster Risk Reduction (UNISDR) defines warning system as a set of capabilities needed for the timely and meaningful generation and dissemination of alert information to individuals, communities and organizations at risk for optimal preparedness and response and at the appropriate time to reduce the likelihood of injury and death [7]. Early warning and timely response play a major role in reducing the vulnerability and mortality caused by disasters and in enhancing the resilience of communities [8]. Deploying appropriate framework and model with the most optimal elements of the warning system can play a significant role in reducing the risk of disasters. In addition, development of warning system modeling will improve system performance [4]. Until now, different models of waning systems with single- or multi-hazard approaches have been developed at various levels [4, 8, 9]. For example, the United Nations Office for Disaster Risk Reduction has proposed a four-element platform including risk knowledge, monitoring, warning dissemination and response [10] or there are a traditional three-phase model proposed by Villagran [11], and an integrated model that has been proposed by Basher [4]. However, each of these models has strengths and weaknesses and there is no consensus on the models and their essential elements [12]. For example, some models focus on risk identification and decision-making, and some focus on warning and response, and there is rarely a model including all the necessary elements [13].

A UN study on the global multi-hazard warning system, as requested by Annan (2005), also showed that the framework and structure of the warning system need further evaluation and evidence [8]. The Global Warning System Review held in 2006 and the Third International Conference on Early Warning also revealed problems and deficiencies of the warning system from various aspects [10, 14]. Therefore, identifying and evaluating existing models and extracting key elements of the warning system are essential for developing an effective and efficient model. These actions play a key role in policymaking in this area and reduce the negative consequences of disasters. In this regard, this study aims to examine the models, patterns and components of the warning system.

### Research question

1. What are the models, patterns and processes of early warning systems in natural disasters?
2. What are the components of early warning systems in natural disasters?
3. What are the advantages and disadvantages of existing models, patterns and processes?
4. What areas need more research?

### Study registration

This systematic review study will be conducted using the PRISMA protocol (PRISMA-P 2015) [15]. This protocol is developed using the PRISMA protocol checklist and is registered in PROSPERO under the number CRD42018116111 (Additional file 1).

## Eligibility criteria

### Type of study

#### Inclusion criteria

In this research, all English studies and documentation published from 1980 to 2019, that have a specific methodology, including initial studies (i.e., interventional, observational and qualitative studies) and secondary studies (i.e., systematic review, narrative review, and meta-analysis) that incorporate elements and conceptual models of early warning systems in natural disasters will be included.

“Related studies” refer to studies that include a warning system used for any kind of natural disaster or include a multi-hazard warning system.

#### Exclusion criteria

All studies with poor methodology, including commentaries, opinion papers, discussion papers and editorials, studies that are not relevant to the research question, studies that their full text is not available or are written in a language other than English, and studies focusing on man-made disasters such as traffic accidents and chemical, biological, nuclear and explosive events are excluded from the review.

### Type of participants

Studies that their research samples have the following characteristics will be selected:

Countries, communities, governmental and private organizations at the international, national, provincial and local levels that have been involved in natural emergencies and disasters, or have participated in a training or simulation program to improve the performance of the alert system

### Information resources and search strategy

Databases such as PubMed, Web of science, Scopus and Google scholar and specialized websites related to emergency and disaster will be searched for documentation and no restrictions on the type of document will apply.

Initially, the keywords are determined and their synonyms are specified using MESH. Then, English keywords and their combinations will be searched in the aforementioned databases based on title tag, summary and keywords from 1980 to 2019.

The syntax searched in the databases will be as follows:

**PubMed syntax**
- (disaster*[tiab] OR emergenc*[tiab]) AND (model[tiab] OR theory[tiab] OR pattern[tiab] OR package[tiab] OR framework[tiab]) AND (“Early warning system”[tiab] OR Notification[tiab] OR EWS[tiab] OR Alert[tiab])

**Scopus syntax**
- (TITLE-ABS-KEY (disaster*) OR TITLE-ABS-KEY (emergenc*)) AND (TITLE-ABS-KEY (model) OR TITLE-ABS-KEY (theory) OR TITLE-ABS-KEY (pattern) OR TITLE-ABS-KEY (package) OR TITLE-ABS-KEY (framework)) AND (TITLE-ABS-KEY (“Early warning system”) OR TITLE-ABS-KEY (notification) OR TITLE-ABS-KEY (EWS) OR TITLE-ABS-KEY (alert))

**Web of science syntax**
- (TS=(disaster*) OR TS=(emergenc*)) AND (TS=(model) OR TS=(theory) OR TS=(pattern) OR TS=(package) OR TS=(framework)) AND (TS=(“Early warning system”) OR TS=(notification) OR TS=(EWS) OR TS=(Alert))

## Study registration

### Selection process

After searching all the databases, the studies will be inserted in the EndNote software. Then the duplicate studies will be deleted. Moreover, the manual search method will be applied in the EndNote software to find duplicates. In the next step, the titles and summaries will be reviewed to find relevant studies. Subsequently, two experienced scholars specializing in the field independently study the full text of the articles. Any potential disagreement between the two reviewers will be resolved through a group discussion. And, in case of further disagreement, a third reviewer will be invited to solve the problem. In addition, to find other related studies, the snowball sampling technique will be used and accordingly the references cited in the reviewed articles will be taken into account. The corresponding author of the reviewed studies will also be requested to report on other relevant studies being completed, in case they know any. Important and credible journals related to this field will also be manually checked to find relevant articles published during the past 10 years. Furthermore, the reference books and legal documentation will also be reviewed to find relevant cases.

### Data collection process

Once the text search process is complete, the researchers will extract and collect data from the full text of the studies. Each person will extract data based on a pre-designed form. The variables include study methodology, hazard type, level and magnitude of the model (i.e., local, regional and national) and the target group of the model (i.e., organization, community), model elements and model strengths and criticisms.

### Risk bias

Given the inclusion of studies with different methodologies, no unique tool for evaluating the methodological quality of studies can be used at this stage. Therefore, in order to evaluate the quality of the included studies, depending on the type of study, the quality assessment tool presented in the Strengthening the Reporting of Observational studies in Epidemiology (STROBE) checklist will be used. At this stage, each model will be evaluated separately by two independent researchers in accordance with their appropriate assessment tools. Any disagreement between the two researchers concerning the quality of the studies will be resolved through the CONSENSUS method and in case of further disagreement at this stage, a third researcher will be asked to comment on the quality of the study.

### Dealing with “missing” data

In case of having difficulty with collecting data from the full-text articles, the corresponding author of the study will be contacted via email to submit the desired data.

### Data analysis

The present study will examine the existing models and elements of the early warning system. The existing models will be compared in terms of elements, relationships between them, and categorized by the type of hazard and the level of the disaster.

### Ethics

This study was approved by the Ethics Committee of the University of Social Welfare and Rehabilitation Sciences of Tehran and has been registered under the registration code: IR.USWR.REC.1397.082.

## Discussion

There are currently different models of early warning systems with different elements depending on the hazard or context [4, 11]. This systematic review provides the specifics of the warning models and their components used in hazards in the context of different communities. It will also highlight the combination of findings, features and limitations of the models. In the initial search, several studies with relevant keywords are entered and then their quality will be evaluated by two raters.

Recognizing different early warning models, their patterns and constituents and examining their strengths and weaknesses seem essential for developing a comprehensive model that includes critical elements. This study is the first step in the development of a comprehensive model of warning system in emergency and disaster, resulting in the recognition of early warning models, their structures, elements and their interactions that ultimately lead to establishing a new approach of warning system and its structure for executives and policymakers to plan and enhance the risk management process. Due to the comprehensiveness of the methodology, the present study can be used to develop an operational model to optimally manage emergencies and disasters, respond to them in a timely manner and reduce their consequences.

## Supplementary information

**Additional file 1.** PRISMA-P + checklist. This checklist has been adapted for use with systematic review protocol submissions to Biomed Central journals from Table 3 in Moher D et al: Preferred reporting items for systematic review and meta-analysis protocols (PRISMA-P) 2015 statement. Systematic Reviews 2015 4:1.

## Data Availability

Data is primarily derived from peer-review publications in the public domain, which may be subject to copyright. We will contact authors for additional information where relevant data is not available in publications. The datasets generated and/or analyzed during the current study are available from the corresponding author on request.

## Abbreviations

EWS: Early warning system
UNISDR: United Nations office for Disaster Risk Reduction
